# A novel enzyme-assisted approach for efficient extraction of Z-ligustilide from *Angelica sinensis* plants

**DOI:** 10.1038/s41598-017-10004-x

**Published:** 2017-08-29

**Authors:** Xin-Guo Zhang, Ying Lu, Wen-Na Wang, Zi-Yu Liu, Jin-Wen Liu, Xiao-Qian Chen

**Affiliations:** School of Life Science and Engineering, Lanzhou University of Technology; Key Laboratory of Screening and Processing in new Tibetan medicine of Gansu Province, Gansu, 730050 P.R. China

## Abstract

Endophytes coexist with plants, in part, due to cellulase that allow saccharification of plant cell walls. The cellulase enzymes found in naturally occurring endophytes may exhibit stronger activity and more specificity than commercially available cellulase for enzyme-assisted extraction of compounds from medicinal plant materials. In order to identify endophytes with high cellulase activity, we screened endophytes taken from different parts of *Angelica sinensis* using the Congo red staining method. We identified three strains with higher cellulase activity. Of the 3 strains identified, No.Lut1201 increased the yield of extracted Z-ligustilide 2 fold compared to commercially available cellulase (Ningxia Sunson) using a cellulase-assisted extraction method and traditional extraction methods. Scanning electron microscopy clearly demonstrated that the cellulase extracted from endophytes enhance cell wall polysaccharide degradation as well as Z-ligustilide extraction from Radix *Angelica sinensis* (RAS). The current study provides a new method and ideas of using cellulase of endophytes for improving the extraction of compounds from medicinal plants.

## Introduction

*Angelica sinensis* is an herbaceous perennial plant that belongs to the umbelliferae family and is widely distributed in western China. Radix *Angelica sinensis* (RAS), also known as *Danggui*, is one of the most important of the traditional Chinese medicines^1^. RAS is used to promote blood circulation, for the treatment of menstrual disorders, to modulate the immune system and as a laxative for chronic constipation of the aged and debilitated^2^. Over 70 compounds have been isolated and identified from RAS, including essential oils, organic acids and their esters, polysaccharides, coniferyl polyacetylenes, vitamins and amino acids. Among the compounds identified, Z-ligustilide is thought to be one of the most biologically active components and is often used for quality control and in pharmacokinetic studies of RAS^3–6^. Recent studies have shown that Z-ligustilide is neuroprotective against stroke and Alzheimer’s disease (AD) through multiple mechanisms, including anti-neuroinflammatory effects^7^.

Numerous methods have been used to extract Z-ligustilide from RAS, including sonication extraction (SE), pressurized liquid extraction (PLE) and supercritical fluid extraction (SFE)^8, 9^. However, the use of organic solvents for the recovery of natural products has several drawbacks, including long extraction times, large organic solvent consumption, high energy input, and contamination of the final product with trace amounts of organic solvents–decreasing its quality. In addition, some methods require special instruments, with only limited digestion of cell wall polysaccharides^10, 11^. Thus, the development of an effective and selective method for bioactive compound extraction would be beneficial^12^. Plant cell-wall polysaccharides present a natural barrier against the release of active compounds from different medicinal raw materials under the same extraction conditions. However, cellulase enzymes can be used to break down the structural integrity of the cell wall, increasing the yield of bioactive compounds from intracellular compartments. In contrast, conventional solvent extraction methods only extract the most accessible compounds from plant material^11–13^. Therefore, enzyme-based extraction of bioactive compounds from plants is a potential alternative to conventional solvent based extraction methods and possesses the advantages of being environmentally friendly, being highly efficient and offering a simplified extraction process^12^. Specifically, recent studies on enzyme-assisted extraction have shown it to be faster, to have a higher recovery rate, to reduce solvent usage and lower energy consumption when compared to non-enzymatic methods^11–15^.

Endophytes are microorganisms that asymptomatically invade plant tissues. They can stimulate plant growth and/or provide a defense against pathogen attacks through the production of secondary metabolites and metabolic enzymes. The majority of endophyte species and their metabolic machinery are still unknown^16^. Therefore, we speculated that specific strains of endophytes likely exist with highly active cellulase enzymes that enable them to digest plant cellulose in order to improve their survival. Furthermore, these endophyte cellulases would possess a greater specificity for the plant cell wall compared to commercially available enzymes; therefore, providing superior results during enzyme-assisted plant extractions.

Based on the above considerations, we used carboxymethyl cellulose (CMC) as a carbon source in medium growth plates to screen and isolate cellulase-producing endophyte strains from *A. sinensis*. Furthermore, we conducted feasibility tests of endophyte cell-wall degrading enzymes as a means for improving Z-ligustilide extraction from RAS. To our knowledge, there have been few studies examining specific degrading enzymes used for extraction of bioactive compounds from plants. Our results confirm that in *A. sinensis* there are endophyte strains that produce more specific cellulases compared with the commercially available enzymes. In particular, our study may also help a new method and ideas of using cellulase of endophytes for improving the extraction of compounds from plants.

## Results and Discussion

### Isolation of the endophytes and screening of cellulase-producing microorganisms

**Figure 1:**
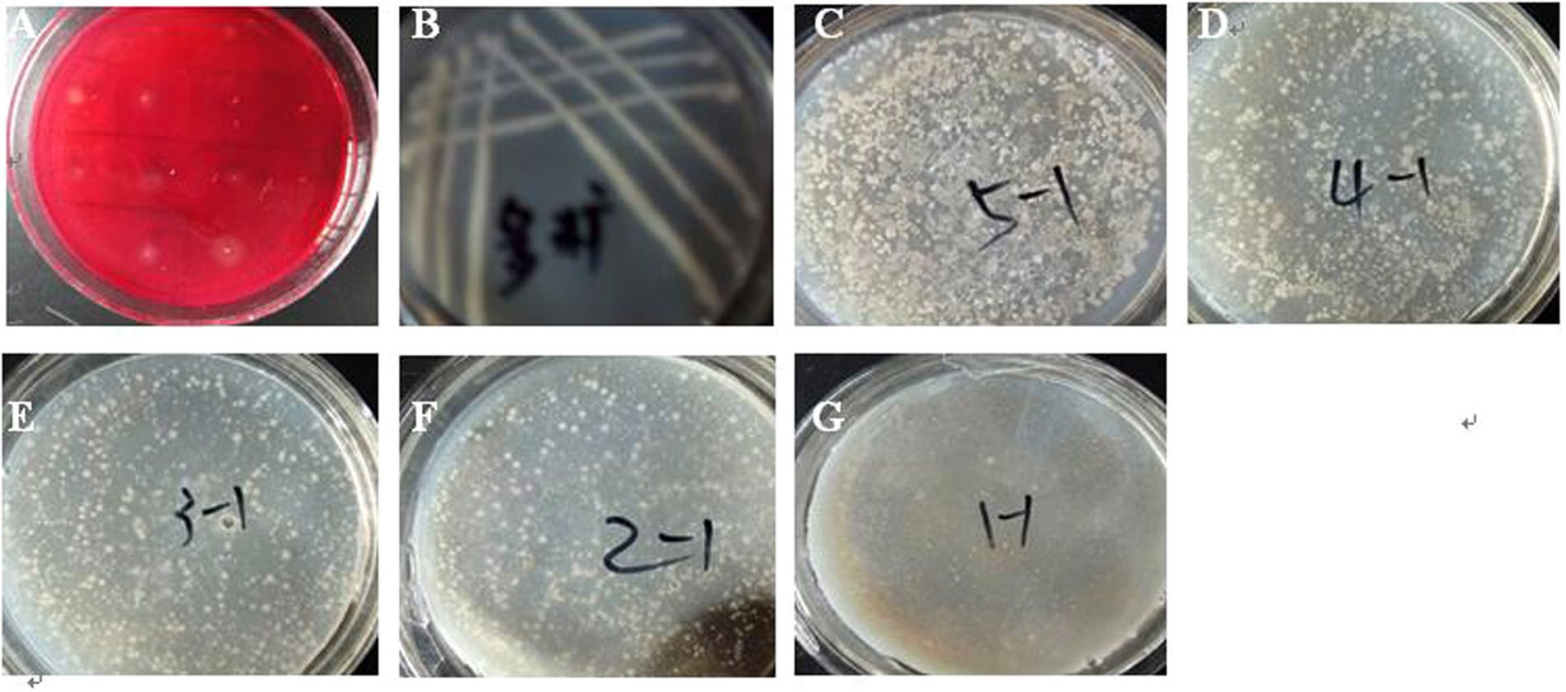
Isolation of endophytes from *Angelica sinensis* and subsequent growth of isolates using Angelica powder and Angelica polysaccharides as the sole carbon source. (**A**) Congo red staining preliminary isolation of cellulolytic endophytes; (**B**) Isolates using Angelica polysaccharides as the sole carbon source to replace the CMC-Na in screening medium A; (**C**–**G**) Isolates using Angelica powder as the sole carbon source to replace the CMC-Na in screening medium A, in here, **C–G** represents that the different C/N ratio(Angelica powder: (NH_4_)_2_SO_4_) is 4:1, 2:1, 1:1, 1:2 and 1: 4, respectively.

Potato Dextrose Agar (PDA) medium is most widely used for isolation of plant endophytes^17–20^. However, for these present experiments, we used a medium that incorporated cellulose as the sole carbon source in order to isolate endophytes capable of producing cellulases. After culturing for more than 7 days, forty-one morphologically distinct endophyte strains were isolated from the leaves, stems and roots of *A. sinensis* samples; most of the endophyte colonies were white on medium A plates. Those isolates were further inoculated in screening medium B, and eighteen isolates were separated using the Congo red staining method (Fig. 1A). Three of the eighteen, having a remarkable clearing zone, were designated No.Lut1102, No.Lut1201 and No.Lut1301, respectively. Their cellulase activity and morphological characteristics are described in Table 1. Compared with the cellulase from other endophytes^16, 18, 19^, these results show that the three endophytes of *A. sinensis* characterized here have a high cellulase activity, and were selected for further study. To our knowledge, this is the first report of endophytes capable of producing cellulases isolated from *A. sinensis*.

**Table 1:** Analysis of morphological characteristics and cellulase activity for three endophytes isolated from *A.sinensis*.

Strains	Plant region	Morphological characteristics	Enzyme activity/(U/mg protein)
No.Lut1102	root	White colonies, oily, smooth, projections, neat edge	523
No.Lut1201	stem	Milky white colonies, oily, colony projections, neat edge	600
No.Lut1301	leaf	filamentous colonies, Green colonies, with rough edges	272

Previously, cellulases were reportedly obtained from different plant endophytes, including *Opuntia ficus-indica Mill (Cactaceae*)^18^ *, Espeletia spp* (a genus unique to the paramo ecosystem, an extreme environment in the Andean mountain range^16^), *Cedrusdeodara, Pinusroxburgii, Abiespindrow* and *Chlorophytumc Omosum* collected from the Kashmirvalley^19^, and seven oleaginous plant species^20^. In these studies, a PDA medium was always used to isolate the endophytes, which were then tested for their ability to produce cellulase. In contrast, here we describe method using CMC agar medium for obtaining cellulase-producing endophytes.

### Effects of enzyme-assisted extraction on Z-ligustilide

Linear regression analysis data for the calibration plot showed a good linear relationship between the response and the concentration in the range of 0.5–100.0 μg/mL; the regression coefficient was 0.9999. These results imply that the method described here is suitable for the routine quantification of Z-ligustilide.

**Figure 2:**
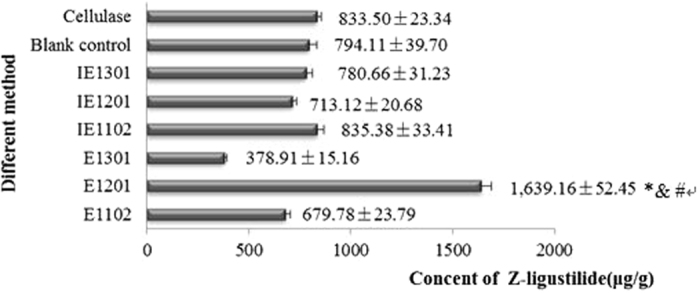
Comparison of different methods for extracting Z-ligustilide from *A. Sinensis*. In here, RAS powder was pretreat with different cellulase for 30 h at 37 °C, and then reflux extraction was performed and the concentration of Z-ligustilide was determined by HPLC. Furthermore, the numbers 1102, 1201 and 1301 represents the three identified endophyte bacterial strains: No.Lut1102, No.Lut1201 and No.Lut1301, respectively. E corresponds to the respective cellulase produced by the bacteria. IE represents the inactivated enzymes of the corresponding reference; blank was without any enzyme treatment using the traditional extraction method. Cellulase represents commercially available cellulase assisted extraction of Z-ligustilide ^*,&,#^p < 0.05 compared with commercially available cellulases, blank and inactivate enzyme control groups.

In order to choose the best enzyme to notably increase the yields of Z-ligustilide, three *A.sinensis* endophytes were compared (No.Lut1102, No.Lut1201 and No.Lut1301) (Fig. 2). Furthermore, in order to eliminate the non-enzymatic processing factors influencing Z-ligustilide extraction and analysis, an extracellular enzyme solution was boiled and used as negative control. As shown in Fig. 2, the differences in the yields of Z-ligustilide among the inactivated enzymes group (IE1102, IE 1201, IE 1301) and blank groups(without any enzyme treatment using the traditional extraction method) were not significant(p > 0.05). These results also demonstrate that boiling the cellulase successfully inactivated the enzyme, while we were successful in using the non-heated, active cellulase to extract Z-ligustilide.

There is little research describing the use of cellulase for extracting Z-ligustilide from RAS. Although the commercially available cellulase increased the extraction yield of baicalein from *Radix Scutellariae Baicalensis* by 1.5 fold compared with the conventional extraction method (data not shown), here we show that the use of commercial cellulase (from *Trichoderma reesei*) resulted in only a weak increase in the extraction efficiency of Z-ligustilide (Fig. 2). The differences in the yields of Z-ligustilide in the commercial cellulase group (833.50 ± 23.34 μg/g) and the blank group (794.11 ± 39.71 μg/g) were not significant (p > 0.0.5). Moreover, some research has reported that commercially available cellulase does not significantly improve the extraction yield of glycyrrhizic acid from *Radix Glycyrrhizae* ^21^, paeonol from *Cortex Moutan Radicis* or paeoniflorin from *Paeonia lactiflora Pall* compared to cellulase prepared using traditional processing methods^22^. Therefore, commercial cellulase is not always suitable for assisted extraction of different plant active ingredients. Furthermore, compared with the conventional reflux extraction method, not all cellulase produced from endophytes increases the yield of Z-ligustilide. In contrast, a 2-fold increase in the yield of Z-ligustilide was obtained using the enzyme prepared from the strain No.Lut1201(1639.16 ± 52.45 μg/g) identified in this study (p < 0.05) (Fig. 2). In comparison, there was an significant decrease(over 2-fold) in the extraction yield of Z-ligustilide using cellulase from strain No.Lut1301(378.91 ± 15.16 μg/g) (p < 0.05). The results indicate that cellulases from the strain No.Lut1301 may contain some unknown enzyme that causes degradation of Z-ligustilide in RAS.

Plants contain primary and secondary cell walls, both of which are fortified by cellulose microfibrils. Cellulose microfibres are crisscrossed within the cell wall with closer alignment and spacing in primary cell walls than in secondary cell walls^23^. Our results show that enzyme extracted from endophytes No.Lut1201 can significantly increase the extraction yield of Z-ligustilide. This could be explained by the superior chimerism between the endophyte cellulases and the plant cell wall that forms a cellulose mesh skeleton^24^. This increase in cellulase activity improves cleavage efficiency of the β−1–4 glycoside bonds, resulting in a 2-fold increase in the yield of Z-ligustilide compared to commercially available cellulases(from *Trichoderma reesei*). Furthermore, this increase in the observed activity seen in cellulase extracted from endophytes is likely due to a natural reliance of the endophytes on their endogenous cellulases in order to survive.

We designed a series of experiments in order to verify endophyte’s symbiotic role with *A.sinensis* plants, as well as the specificity of their cellulose-degrading enzymes. In brief, Angelica polysaccharide and Angelica powder were used as the sole carbon source to replace the CMC-Na in screening medium A. After sterilization, the strain No.lut1201 was used to inoculate the cultures and then incubated at 37 °C for 48 h (see Fig. 1B–G). The results confirmed that No.lut1201 is able to take advantage of Angelica polysaccharides (Fig. 1B) and powder (Fig. 1C–G) as a carbon source for growth. Moreover, using increased proportions of Angelica powder as a carbon source in the medium resulted in a clearly increase in the number of endophytes (Fig. 1C–G); when the content of angelica powder fell to a C/N ratio of 1:1, the growth of endophytes decreased clearly (Fig. 1C–G). These results further suggest that Angelica powder can modulate endophyte cell growth.

### SEM analysis (microscopic morphology of enzyme-treated extraction)

**Figure 3:**
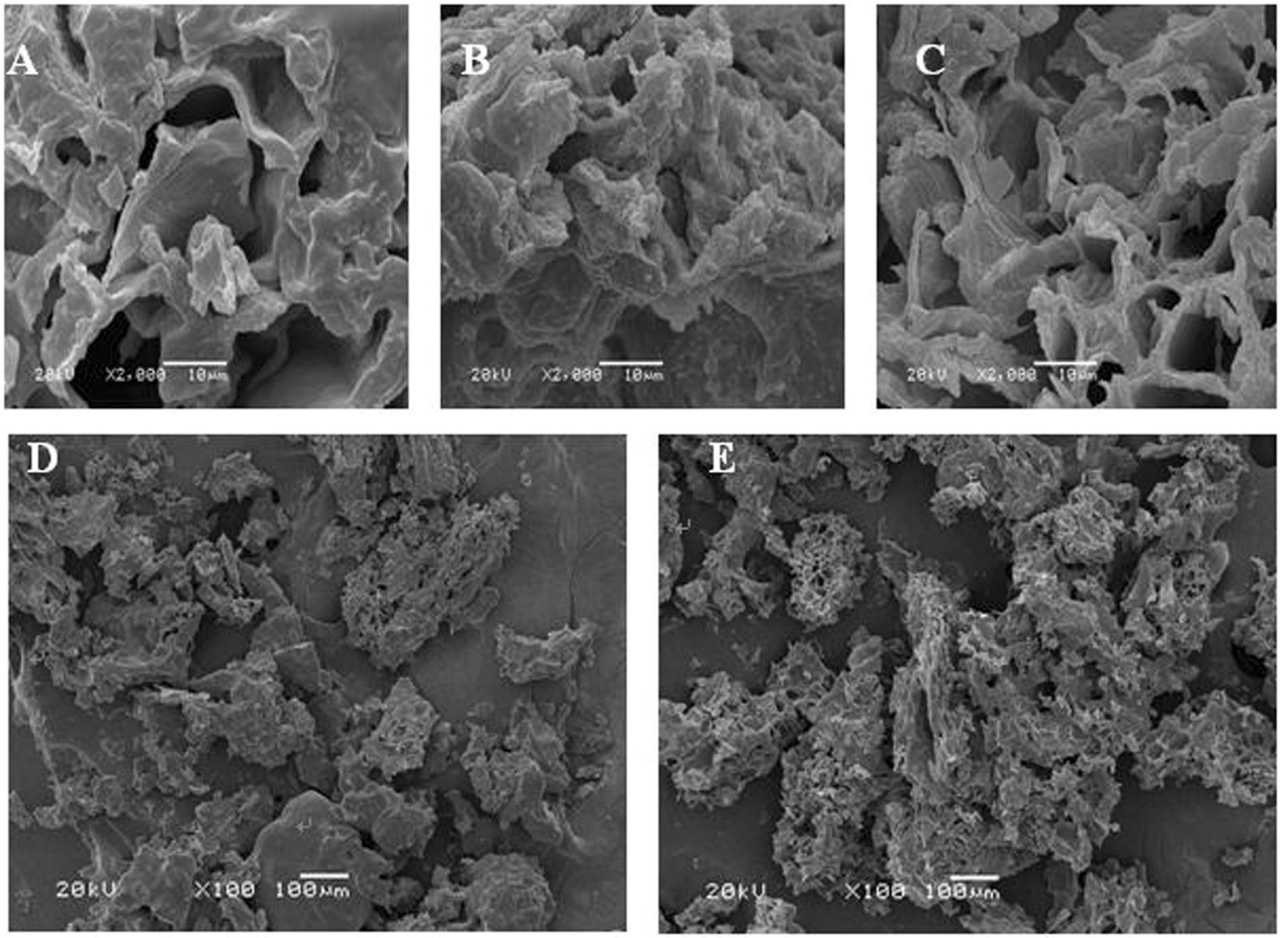
Scanning electron micrographs of *A.sinensis* powder under different treatment processes. (**A**–**C**) A water only, B commercial cellulase and C endophytes cellulase treatment (10 μm, 2000 × magnification), respectively; (**D** and **E**) are commercial cellulase and endophyte cellulase treatment (100 μm, 100 × magnification), respectively.

In order to evaluate the degrading cell wall effects of cellulase treatment on the extraction of Z-ligustilide from RAS, SEM was used to compare the changes in microstructure of the herbal powder, RAS, with and without enzymatic hydrolysis. Figure 3 shows the differences between RAS treated with commercially available enzyme, enzyme from endophytes and non-enzyme treatments. There were no ruptures or significant destruction to the microstructure with commercially available enzyme and with the non-enzyme treatment (Fig. 3A,B). However, after treatment with the endophyte cellulase, the cell wall of RAS became thinner and the microstructure was disorganized (Fig. 3C).

Using low magnification SEM (100μm, 100 × magnification), the significant destruction of RAS cell wall can be seen (Fig. 3D,E). The results of the SEM micrographs suggest that cellulase from endophytes can destroy or weaken the structures of RAS cells walls efficiently, allowing the intracellular active component to be more accessible for extraction.

### Isolation of cellulase and analysis of purified proteins by SDS-PAGE

**Figure 4:**
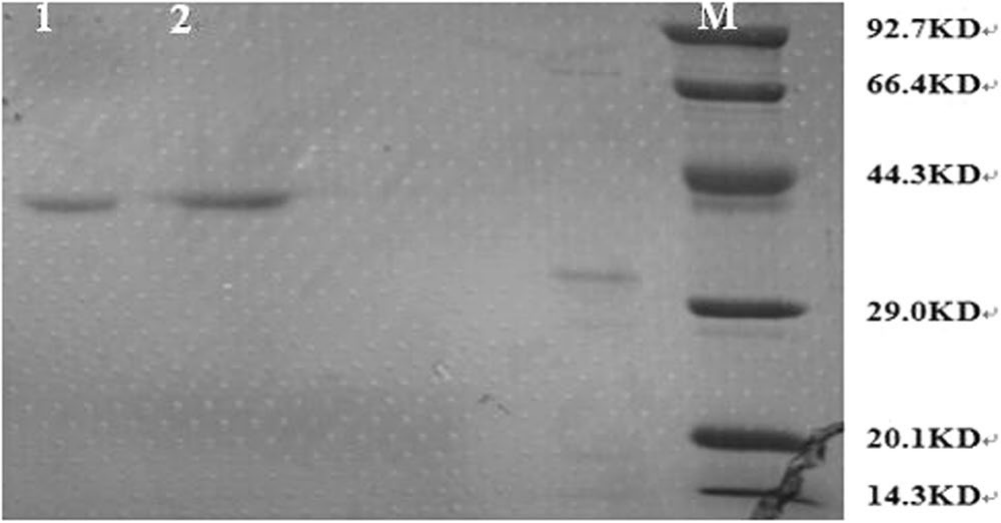
The SDS-PAGE electrophoresis of cellulase from No.Lut1201 using the centrifugation method. Lanes 1 and 2 represent cellulase from strain No.Lut1201; M represents the Standard protein molecular weight marker.

The culture supernatants were collected by centrifugation and analyzed by electrophoresis using SDS-PAGE and coomassie blue staining. The results are shown in Fig. 4, showing a clear, single band, confirming that a high purity cellulase has a molecular weight of about 40 kDa and was obtained using a simple centrifugation process from strain No.Lut1201 supernatant enzyme solution^25, 26^. As reported in the literature, most procedures require a multistage chromatographic purification process containing ion chromatography, hydrophobic exchange chromatography and gel chromatography to obtain a single enzyme, regardless of whether the sample comes from a fungus or bacteria^13, 27, 28^. In the present study, for the first time, we collected a high purity cellulase by using a simple centrifugation process and our results show that endophytes can produce high specificity and high purity cellulase, which could be a possible reason for a significant increase in the extraction yield of Z-ligustilide.

### Identification of endophyte strains

**Figure 5:**
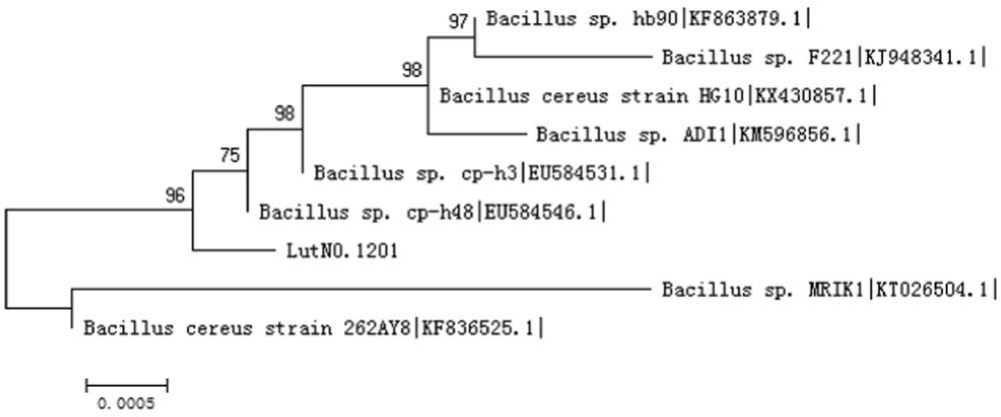
Phylogenetic tree of strain No.Lut1201.

The 16S rDNA sequence is a convenient and universal marker for identifying bacterial species. An alignment based on BLAST analysis revealed that the partial 16S rDNA region of isolate No.Lut1201 corresponds to a *Bacillus sp*. A phylogenetic tree was constructed using maximum-parsimony and neighbour-joining with MEGA 5.0 software (Fig. 5). The numbers at the nodes indicate the level of bootstrap support, based on a neighbor-joining analysis of 1,000 resample datasets; only values >50% are indicated. The bar represents 0.05 substitutions per nucleotide. The topology of the tree was evaluated using a bootstrap analysis with 1000 replications. The highest 16S rRNA sequence similarities between the isolate and type strains of recognized species in the GenBank databases were 99%^24, 29^.

### Effect of differentfactors on the growth and enzyme production of endophytes

**Figure 6:**
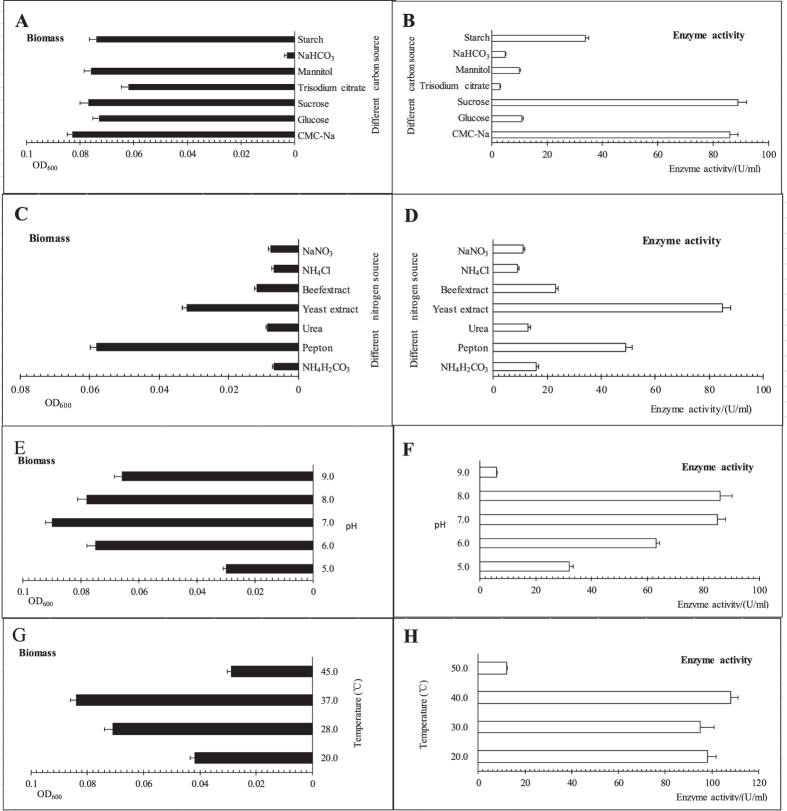
Effect of different factors on the growth and enzyme production of endophytes. (**A**,**B**) CMC-Na, sucrose, glucose, sodium citrate, mannitol, NaHCO_3_ and starch was used as a carbon source for evaluating biomass and enzyme production; (**C**,**D**) (NH_4_)_2_SO_4_, peptone, beef extract, yeast extract, sodium nitrate, ammonium dihydrogen phosphate, urea and ammonium chloride was used as a nitrogen source for evaluating biomass and enzyme production; (**E**,**F**) The growth and enzyme production were measured under a pH = 5.0, 6.0, 7.0, 8.0 and 9.0; (**G**) The growth was measured at 20 °C, 28 °C, 37 °C and 45 °C; (**G**) The enzyme activity was measured at 20 °C, 30 °C, 40 °C and 50 °C.

The effects of different carbon sources, nitrogen sources, pH and temperature on the growth and enzyme production of endophytes are shown in Fig. 6A–H. The results confirmed that No.Lut1201 isolates could grow using different factors, but those factors have different effects on the growth and enzyme production of the strain. As shown in Fig. 6A, an organic carbon source for growth is better than that of inorganic carbon. No.Lut1201 grew best when the carbon source was CMC-Na. When the carbon source was sodium bicarbonate, after adjusting pH to 7.0, we determined that the growth of No.Lut1201 was significantly inhibited, indicating that sodium bicarbonate as a carbon source is not ideal for its growth. Using different organic carbon sources also had a significant effect on the production of cellulase by No.Lut1201 isolates (Fig. 6B). Using sucrose as the carbon source, the cellulase activity is highest, followed by CMC-Na. When the supplied carbon source was glucose, mannitol, sodium bicarbonate or sodium citrate, enzyme activity was significantly inhibited.

As shown in Fig. 6C, an organic nitrogen source for the growth of No.Lut1201 isolates is better than that of inorganic nitrogen. When the nitrogen source was peptone, the strain grew best; when the nitrogen source was inorganic nitrogen, including urea, ammonium chloride, sodium nitrate and ammonium dihydrogen phosphate, the results showed a similar poor growth of the strain. Moreover, when the nitrogen source was from a yeast extract, cellulase activity was highest (Fig. 6D). When the supplied nitrogen source was beef extract, sodium nitrate, ammonium dihydrogen phosphate, urea, ammonium chloride or sodium citrate, enzyme activity was significantly inhibited.

As shown in Fig. 6E, within the range of pH 5.0–8.0, the growth of No.Lut1201 increased with pH value. At pH 7.0, the cell growth peaked, after that with any increase in pH, cell growth decreased gradually; a partial acidic or partial alkaline environment caused an obvious inhibition in growth. Moreover, as seen from Fig. 6F, at pH 7.0–8.0, cellulase activity was highest. At pH < 5.0 and pH > 9.0, enzyme activity was significantly inhibited.

As shown in Fig. 6G, the No.Lut1201 strain grew in the range of 20 °C–50 °C, with the most optimum growth temperature occurring at 37 °C. At 20–40 °C, the enzyme activity was not significantly influenced; however, when the temperature was higher than 50 °C, enzyme activity declined. In this present study, the optimum enzyme activity temperature was 40 °C (Fig. 6H).

### Characterization of cellulase

**Figure 7:**
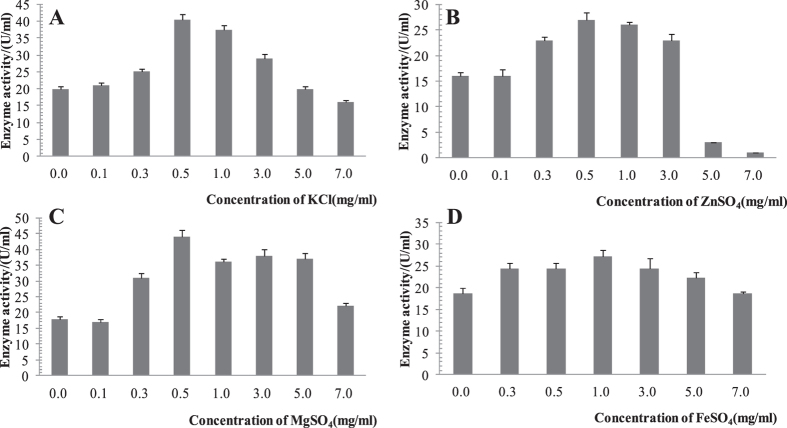
Characterization of cellulase enzymes in the presence of metal ions. (**A**) potassium ion (**B**) zinc ions (**C**) magnesium ion and (**D**) iron ion.

The characteristics of cellulase from endophyte No.Lut1201 are summarized in Fig. 7. Figures 7A–D shows that different metal ions have different effects on the cellulase activity with a bell-shaped distribution. At low concentrations, increases in potassium ion, magnesium ion and iron ion concentration have some effect on cellulase activity. 05.–1.0 percent in potassium, magnesium, and iron ion concentrations within the range of 0.5–1.0% resulted in the strongest activation effect on cellulase from No.Lut1201. However, at high concentrations, these metal ions had a significant inhibitory effect. Similarly, at low concentrations, zinc did not have an obvious effect on cellulase activity, but resulted in a strong inhibitory effect at high concentrations.

## Conclusion

To the best of our knowledge, there have been few studies examining specific degrading enzymes used for extraction of bioactive compounds from plants. Endophytes are a class of microorganism that lives symbiotically with the plant host for a long time. Therefore, it is possible that the endophyte cellulase enzymes enable the saccharification of plant material to promote survival. In addition, cellulase from endophytes differs in its activity compared to commercially available enzymes, with a greater specificity for degradation of the plant cell wall. Therefore, endophyte cellulases are ideal for use in the enzyme-assisted separation of the active ingredients in plants.

In the current study, we confirmed our hypothesis that endophytes can produce exclusive cellulase with increased activity compared to commercially available enzyme. The endophyte isolate No.Lut1201 can also grow in medium with Angelica powder and Angelica polysaccharide as a carbon source. Using SDS-PAGE electrophoresis, we confirmed here for the first time that endophytes produce high purity cellulase. Moreover, endophyte cellulase rather than whole cell significantly increases the yield of Z-ligustilide from RAS 2.0 fold. Therefore, our results offer a new method and ideas for biological methods to extract active ingredients from traditional Chinese medicine.

## Materials and Methods

### Plant material

The dried RAS were bought from Minxian County of Gansu Province of China and ground into powder. Sifters were used to maintain the particle size between 280 and 600 μm. Moreover, endophytes were isolated from fresh *A. sinensis* plant samples collected from the wild. Dr. Lin Yang from the Lanzhou University of technology identified the samples as RAS and *A. sinensis*.

### Chemicals and enzymes

Z-ligustilide was purchased from the Biotechnology Company of ChenDu ChromaBio (purity > 98%, Beijing, China). Chromatographic grade methanol was obtained from Yuwang Chemical Reagents Company (Shandong, China). Cellulase was purchased from the Ningxia Sunson Cellulase Preparation Plant (from *Trichoderma reesei*, ≥11 U/mg). All other chemicals regents were of analytical reagent grade and purchased from GuangFu Reagent Company (TianJin, China). The deionized water was replaced with purified water purchased from Wahaha Group Co., Ltd and filtered through 0.22 μm membranes (Hangzhou, China).

### Instruments and chromatographic conditions

The HPLC system (JASCO, Kyoto, Japan) consisted of a PU-2086 pump, a 6-valve sample injection port fitted with a final volume sample loop of 20 μL and a UV-2075 detector. The chromatographic separation was achieved on a reversed-phase C_18_ column (SinoChrom ODS-BP, 4.6 mm × 250 mm, 5.0 μm, Elite, Dalian, China) that was protected by a C_18_ guard column (ODS 4.6 mm × 10 mm, 15.0 μm, Elite, Dalian, China).

HPLC chromatographic conditions for determination of Z-ligustilide were as follows: the mobile phase was composed of a mixture of methanol-water (60:40, v/v), filtered through a 0.45 μm filter under vacuum and degassed prior to use. The flow-rate was 1.0 mL/min. Ultraviolet detection wavelength was set at 327 nm and the column temperature was kept at 25 °C^29^. An accurately weighed Z-ligustilide standard (1 mg) was dissolved in a 1 mL mobile phase solution to prepare a stock standard solution (1000 μg/mL). The standard stock solution was diluted with the mobile phase to obtain working solutions of 0.5, 1, 5, 10, 25, 50 and 100 μg/mL. The standard curve of Z-ligustilide was analyzed by using the linear least squares regression equation derived from the peak area^9, 30^.

### Isolation and culture of endophytes

The samples were verified as healthy, and while still fresh the roots, stems and leaves were washed thoroughly in running tap water for 5–10 min. Sections of leaves, stems and roots of 1 cm in length were surface-sterilized in the following sequence: 75% ethanol for 1 min, 2% NaClO solution for 3 min (leaves), 4 min (stems), or 5 min (roots), respectively; then rinsed six times in sterile distilled water to remove surface sterilizing agents. The sterile water from the final rinse was collected to evaluate the sterilization efficiency^31^. The surface-sterilized plant samples were ground in an ice bath under aseptic conditions and plated on the medium A plates {CMC-Na(Mw, 1200) 5 g, (NH_4_)_2_SO_4_ 1 g, MgSO_4_ 0.5 g, KH_2_PO_4_ 1 g, Na_2_HPO_4_ 1 g, agar 20 g and 1000 mL water, pH 7.0}. Each plate was incubated at 30 °C for 7–10 day. In addition, 0.1 mL of the sterile distilled water used during the final rinse was placed in beef extract peptone medium(beef extract peptone medium (beef extract 5 g, peptone 10 g, NaCl 5 g, agar15 g and 1000 mL water, pH 7.0) and cultivated in the same conditions as a control for evaluating the sterilization efficiency^31^.

### Screening of cellulase producing endophytes

We screened for cellulase producing endophyte isolates by streaking cellulose Congo-Red agar plates. Briefly, the isolated endophyte colonies previously determined to have different morphologies were inoculated to the solid screening culture medium B (CMC-Na 20 g, (NH_4_)_2_SO_4_ 1 g, MgSO_4_ 0.5 g, KH_2_PO_4_ 1 g, Na_2_HPO_4_1g, Congo-Red 0.2 g, agar 20 g and 1000 mL water, pH 7.0). The use of Congo-Red as an indicator for cellulose degradation in an agar medium provides the basis for a rapid and sensitive screening test for cellulolytic microorganisms^32^. Because of Congo-Red’s high affinity for cellulose, colonies showing less Congo-Red staining were identified as positive cellulose-degrading bacterial colonies. Cellulase activity was determined by measuring the ratio of the colony diameters to clear zone diameters^32, 33^. The endophyte colonies with high cellulase activity were then selected and used in subsequent experiments.

### Preparation of cellulaseh

The cellulolytic microorganism isolates were used to inoculate 100 mL liquid medium C(CMC-Na 10 g, peptone 10 g, NaCl 5 g and 1000 mL water, adjust the pH to 7.0) at 37 °C. After 30 hours of culture, the broth was separated by centrifugation (8,000 g, 10 min) at 4 °C and the supernatant was collected. The purity of the cellulases was analyzed by sodium dodecyl sulfate-polyacrylamide gel electrophoresis (SDS-PAGE) with a 10% acrylamide gel and 5% condensing gel in a Mini-Protein II electrophoresis unit (Bio-Rad). The gel was stained with 0.25% Coomassie brilliant blue R-250 (Aldrich, USA). Running and staining procedures were performed according to the supplier’s protocol^34^. The protein concentration was determined by the Bradford method with bovine serum albumin as a standard^34^.

### Cellulase activity assay

Cellulase activities were determined as previously reported^35, 36^. In brief, the broth was centrifuged at 7104 g/min for 10 minutes and then the cellulase activity was measured directly in the supernatant as following: the enzyme (0.2 ml) and 1.0% (w/v) CMC (1.0 ml) were incubated in 0.1 M sodium phosphate buffer (pH 7.0) at 37 °C for 30 min. A boiled enzyme extract served as a blank control and we used d-glucose(0.01–0.06 μg/ml) as a standard. We then added 3–5, dinitrosalicylic acid (DNS) 0.03% (w/v) (1.0 mL), and followed by boiling the samples for 5 min to stop the reaction. The tubes were cooled down to room temperature and were diluted with distilled water (4.0 ml). The presence of reducing sugars was quantified by measuring absorbance at 540 nm. One unit (U) of cellulase activity was defined as the amount of enzyme that produced 1 μmol reducing sugar/min under the above conditions^35, 36^. The results allowed us to select endophyte strains producing enzymes with high cellulase activity.

### Reflux Extraction of Z-ligustilide

RAS powder were transferred (5 g, 0.2–0.6 mm) into a reflux extractor and then 100 mL of 70% ethanol added to perform extraction at azeotropic point(78 °C). The extraction was terminated when the extract turned clear. The residue was then filtered through Whatman filter paper. We repeated the extraction and filtration processes twice. The filtrate was then collected and concentrated in a rotary evaporator. The syrup was reconstituted with methanol and filtered through a membrane filter (0.45 μm pore size) before analysis by HPLC as above described^9, 30^.

### Enzyme-assisted extraction and pretreatment

Extracellular cellulase from *A. sinensis* endophytes was separated and purified as described above, then accurately quantified and dispersed in a pH 7.0 phosphate buffer solution to obtain enzyme solutions of 15 U/mL. The commercial cellulase used in these experiments was dissolved in a pH 7.0 phosphate buffer solution and used as a positive control to ensure that the final enzyme activity from the cellulases extracted from *A. sinensis* endophytes was within a similar range of 15 U/mL. The extracellular cellulase extracts were then divided into two parts: one was boiled for use as a negative control and the second was used to measure enzymatic activity. RAS powder (5.0 g dried and grounded) was added to 100 mL enzymatic solution, then mixed and adjusted to pH 7.0. The solution was then shaken in a reciprocating shaker for 30 h at 37 °C. Then, the solution was filtered through Whatman filter paper and the residue was collected into conical flasks. Subsequently, reflux extraction was performed and the concentration of Z-ligustilide was determined by HPLC as described above^9, 30^.

### Scanning electron microscopy

We used scanning electron micrographs (SEM, Quanta-200) to evaluate the effect of cellulase from different sources on the structural integrity of RAS samples. The RAS samples were treated with commercially available cellulase, endophyte-originated cellulase or a non-enzyme control. The samples were fixed on adhesive tape, coated with gold then examined under high vacuum conditions at an accelerating voltage of 20 kV (10 μm, 2000 × magnification; 100 μm, 100 × magnification)^11, 13^.

### Identification of bacterial strains

Genomic DNA was extracted from LutNo.1201 using Ezup Column Bacterial Genome DNA Extracting Kit (Sangon Biotech, Shanghai, China) according to the manufacturer’s protocol. The 16 S rDNA from the extracted DNA was amplified by PCR using primer 27 f (5′AGAGTTTGATCMTGGCTCAG) and primer 1492r (5′GGTTACCT TGTTAC GACTT). Polymerase chain reaction (PCR) was carried out in 50 μL reaction mixtures containing 5 μL 10 × PCR Buffer, 4 μL 2.5 mmol/L dNTPs, 1 μL 5 μmol/L Forward Primer, 1 μL 5 μmol/L Reverse Primer, 0.5 μL Taq enzymes, 1 μL Template DNA and adds ddH_2_O to 50 μL. The conditions used for thermal cycling were as follows: denaturation at 94 °C for 3 min, followed by 32 cycles of denaturation at 94 °C for 30 seconds, annealing at 56 °C for 30 seconds and an extension at 72 °C for 50 seconds. At the end of the cycles, the reaction mixture was kept at 72 °C for 7 min and then cooled to 4 °C^37^. PCR products were detected with 1% gel electrophoresis and were send to analyze by Sangon Biotech (Shanghai, China). The 16S rDNA gene sequences were BLAST searched against the GenBank database (http://www.ncbi.nlm.nih.gov/). The sequences determined and reference sequences downloaded from GenBank were aligned using multiple-sequence alignment software CLUSTAL X version 1.81. Phylogenetic trees were constructed with the molecular evolutionary genetics analysis software MEGA version 3.1 program.

### Factors influencing endophyte growth and cellulase enzyme activity

Media containing different carbon sources, nitrogen sources and pH were prepared using medium A. Specifically, the CMC-Na (w/v, 0.5%) was replaced with sucrose, glucose, sodium citrate, mannitol, NaHCO_3_ and starch; (NH_4_)_2_SO_4_ (w/v, 0.1%) was replaced with peptone, beef extract, yeast extract, sodium nitrate, ammonium dihydrogen phosphate, urea and ammonium chloride; the pH was adjusted to 5.0, 6.0, 7.0, 8.0 and 9.0 with HCl and NaOH using a pH meter, respectively. The growth medium (100 mL) was formulated in 500 mL Erlenmeyer flasks and sterilized. The sterilized medium was then inoculated with strain No.Lut1201 (1%), followed by shaking at 37 °C for 72 h. A small aliquot of the broth (1.0 mL) was then distilled with 0.9% saline for analysis of biomass by measuring absorbance at 600 nm, using water as a reference. At the same time, the cellulase activity in the supernatant was determined as described above.

In order to evaluate the effect of temperature on the growth of endophytes, the cellulolytic microorganism isolate No.lut1201 was inoculated in 100 mL liquid medium C at temperatures of 20 °C, 28 °C, 37 °C and 45 °C, the biomass was then measured after 30 h. Moreover, in order to evaluate the effect of temperature on enzyme activity, 100 mL cellulase broth supernatant was incubated at temperatures of 20 °C, 30 °C, 40 °C and 50 °C for 30 min; the enzyme activities were then measured as described above. All experiments were repeated in parallel three times.

### Characterization of cellulase enzymes

In order to evaluate the effect of different metal ions on enzyme activity, the cellulase broth supernatant was added to KCl, FeSO_4_, ZnSO_4_ or MgSO_4_ solutions to reach a final concentration of 0, 0.01%, 0.03%, 0.05%, 0.1%, 0.3%, 0.5% and 0.7%; enzyme activity was then measured as described above.

### Statistical analysis

Mean values of all data were obtained from triplicate experiments and analyzed by one-way analysis of variance (ANOVA) followed by Duncan’s multiple range test using the SPSS17.0 software. Statistically significant differences between control and experimental groups were defined as p < 0.05.
